# A machine learning approach to healthcare needs and barriers using the 100% Community Survey of access to SDOH services

**DOI:** 10.3389/fpubh.2025.1659322

**Published:** 2025-09-10

**Authors:** Karikarn Chansiri, Julie S. McCrae, Katherine Ortega Courtney, Dominic Cappello

**Affiliations:** ^1^Chapin Hall Center for Children, Chicago, IL, United States; ^2^Anna, Age Eight Institute, New Mexico State University, Las Cruces, NM, United States

**Keywords:** healthcare access, social determinants of health, health disparities, machine learning, 100% Community

## Abstract

**Background:**

Access to health care is a key social determinant of health, yet individual experiences of need and barriers—especially in rural and racially diverse regions—are often overlooked. Traditional models may miss complex sociodemographic and household patterns. This study applies machine learning (ML) to examine healthcare needs and access barriers among adults in New Mexico, a diverse state with high service needs.

**Objectives:**

(1) Identify predictors of self-reported healthcare needs across medical, dental, and mental health domains; (2) determine factors and reasons linked to access barriers; (3) compare performance across seven ML algorithms; and (4) generate interpretable insights to inform interventions.

**Methods:**

We analyzed survey data from 9,099 adults across 13 New Mexico counties (2019–2024). Predictors included sociodemographic, geographic, and household factors. Models—spanning linear, tree-based, kernel-based, and neural networks—were evaluated using recall, F1-score, and area under the precision-recall curve. Interpretability tools included SHAP, partial dependence plots, and permutation importance.

**Results:**

(1) Predictors varied by domain. Mental health needs were linked to younger age, low income, limited family support, and being female. Dental needs were highest among higher-income White parents; medical needs were tied to larger households and parenting status. Family support consistently reduced barriers. (2) Common barriers included cost, wait times, and provider shortages. Hispanic respondents reported fewer mental health barriers. (3) Neural networks and tree-based models performed best (recall up to 0.99). (4) Interpretability methods revealed complex, nonlinear predictor patterns.

**Conclusion:**

ML models revealed complex, domain-specific patterns of need and access, highlighting the limitations of one-size-fits-all approaches. Community-based initiatives like 100% Community can leverage these insights to target structurally excluded populations and strengthen local support systems. Hyperlocal planning, state-level policy reform, and family-centered interventions are essential to addressing healthcare disparities in high-need settings.

## 1 Introduction

Healthcare in the United States remains among the most inaccessible in the developed world, despite high spending and decades of reform efforts (1). A comparative study using 71 performance metrics—including expert surveys and data from the World Health Organization and Organization for Economic Co-operation and Development—found that U.S. healthcare ranks poorly, and the population is, on average, “sicker,” with higher rates of chronic conditions and disease burden compared to peer nations (1). The Affordable Care Act and more recent strategies such as value-based care models and social needs screening have made some progress (2, 3), but systemic inequities persist. As of 2017, 43% of publicly insured adults delayed or avoided necessary care, and 24% missed preventive checkups (4). Nationally, 15–17% of adults with chronic conditions still report difficulty accessing care (5), while over half of U.S. youth with mental health needs receive no services at all (6).

These gaps are especially pronounced in low-income, racially diverse, and rural communities (7, 8). In New Mexico, for example, healthcare access is challenged by provider shortages and demographic factors, including a high proportion of Hispanic and Native American residents (9, 10). Identifying individuals who perceive unmet needs and barriers to care in dental, mental, and physical health is critical to designing equitable interventions (11–13). Unlike clinical outcomes, perceptions of need and access offer insight into social determinants that influence healthcare use but are not easily captured in medical records (11–13).

Perceived healthcare needs—defined as the recognition of a personal need for care—arise from a combination of Sociodemographic, geographic, and household factors (11–13). Across care domains, disparities are particularly evident in dental care, where ethnic and racial minority and low-income groups report both higher rates of untreated needs and lower utilization of services (14). Similarly, Hispanic, Black, and Native American communities experience greater psychological needs and chronic illness yet remain underserved in preventive mental health care (15, 16). A longitudinal study showed that low-income Black Americans develop multimorbidity earlier than White or higher-income peers, likely increasing awareness of medical health needs (16). Gender also plays a role: women—especially older women—are more likely to report both medical and mental health concerns (17). These disparities are often compounded in rural areas, where provider shortages, poverty, and racial disparities intersect to produce particularly high perceived need (18–20).

Like healthcare needs, barriers to care are shaped by multiple, overlapping factors, including income, race and ethnicity, gender, family dynamics, and geographic access (19, 20). Although self-reported barriers may lack clinical precision, they offer important insights into patients’ lived experiences and decision-making processes (21). Income remains a dominant factor; a national survey found that 95% of adults who delayed or skipped care cited low-income (20), and by 2022, more than half of low-income adults had forgone care due to financial barriers across all domains—medical, dental, and mental health (22).

Racial and ethnic minority groups disproportionately report structural barriers such as provider shortages, discrimination, and mistrust—especially among young adults (22, 23). These barriers frequently intersect with income-related challenges. For example, younger Hispanic and Black adults more often cite financial barriers than White counterparts, who are more likely to cite time-related issues (23). Gender disparities also emerge: younger women face cost barriers specific to reproductive care, and in non-Medicaid expansion states, they are more likely to be uninsured during childbearing years (24). Geographic isolation further limits access, particularly in rural settings like those across New Mexico, where mental and dental care resources are especially scarce (14, 20). Social support—such as help with transportation or caregiving—has been shown to reduce perceived barriers and improve continuity of care (20).

Existing studies have explored predictors of perceived needs and barriers to care primarily within sectors, assessing only one of the dental, medical, or mental healthcare domains, rather than taking a holistic approach. In addition, most research has relied on traditional statistical methods (9, 10), that do not sufficiently allow for nonlinear or high-dimensional patterns that could capture more complex, unique relationships in need for and access to care. Machine learning (ML) is a powerful alternative in that it is able to flexibly model intricate interactions in demographic, economic, and household factors beyond linear relationships and specific to each sector of interest (25, 26).

There is growing interest in ML for healthcare applications, but to date studies are limited in community (compared with clinic or hospital) settings and focused on clinical outcomes, with sparse use of ML to understand needs and access to care from the perspective of consumers which can inform tailored interventions (27–29). When used, ML studies have generally focused on single domains, single algorithms, or demographically homogenous samples. For example, a deep learning study using Medical Expenditure Panel Survey data (*N* = 25,200) predicted unmet dental care needs with high accuracy (recall = 77.8%, precision = 82.9%, accuracy = 82.6%) but was limited to a small proportion (6.6%) reporting need and did not explore mental or physical health domains (28). Another study in Virginia applied multiple algorithms to predict behavioral health diagnoses, a consequence of unmet needs and barriers to care, among Black patients and found that model performance varied by algorithm and outcome (24). Gradient boosting outperformed others (accuracy = 0.93, precision = 0.790, recall = 0.709), suggesting that different ML models may yield different insights depending on context. Yet, that study relied on clinical data and did not directly examine perceived need or access barriers.

In sum, this study addresses two gaps in our understanding of perceived need and access to health care. First, there is a lack of information about access to dental, medical, and mental health care holistically as generated in community-based settings where individuals are seeking care, particularly in very low-resourced communities. Second, analytic models investigating need for and access to care lack ML approaches that allow for greater complexity in non-linear relationships. We address these limitations by using multiple ML algorithms and interpretable techniques to predict perceived barriers and needs in dental, medical, and mental healthcare, using survey data from 13 diverse counties in New Mexico. By focusing on Sociodemographic, geographic, and household factors, our approach aims to inform more equitable and targeted public health strategies. We propose four aims:

- Aim 1: Identify the most important predictors of perceived healthcare needs and access barriers in underserved communities.
- Aim 2: Compare ML model performance across medical, dental, and mental health domains.
- Aim 3: Describe the most commonly reported reasons for barriers to care.
- Aim 4: Generate interpretable, actionable insights to guide targeted interventions and policy.

## 2 Method

### 2.1 Data source

We conducted a cross-sectional analysis using data from the 2019–2024 100% Community Survey. This survey systematically collects comprehensive, self-reported information from residents across 13 counties in New Mexico, including Bernalillo, Catron, Curry-Roosevelt, Guadalupe, Otero, Rio Arriba, San Juan, San Miguel, Santa Fe, Socorro, Taos, Valencia, and Doña Ana. Participants (*N* = 9,099) were adults aged 18 years or older residing within the 13 specified counties listed above.

### 2.2 Survey instrument

The 100% Community Survey included comprehensive questions designed to assess a broad set of perceived healthcare needs and barriers encountered when accessing dental, medical, and mental health services. Predictors used in this study encompassed multiple domains, including sociodemographic, geographic, and household factors.

Demographic variables included age (categorized from 18–24 to 55 years or older) and gender (originally including woman, man, nonbinary, and prefer not to answer). Due to small subgroup sizes, gender was recoded as a binary variable (woman vs. man) to ensure model stability. Race and ethnicity were operationalized as separate binary indicators for each group: Asian, Black, Hispanic, Native American, and White. Economic indicators included annual household income and education level, both treated as ordinal categorical variables. Income was categorized into brackets ranging from less than $10,000 to more than $70,000, and education ranged from less than high school to graduate/professional degree.

Geographic information included county of residence, with each of the 13 counties represented as a binary indicator. Household-level predictors included total household size (ranging from 1 to 7 or more), parental responsibility for children under 18 (yes/no), number of children under age 5, and number of children aged 5–18. The two child-related variables were measured on a 5-point ordinal scale (0, 1, 2, 3, 4 or more) and treated as continuous variables in the analysis to capture general trends in household composition. Perceived family support was measured using a Likert-style item asking participants to rate the extent to which they had extended family support nearby, with responses ranging from 1 (strongly disagree) to 5 (strongly agree).

Outcome variables consisted of six binary indicators reflecting whether respondents reported healthcare needs or access barriers in the past year across the medical, dental, and mental health domains. For respondents who indicated experiencing barriers to care, a follow-up question asked them to select the specific challenges encountered from a predefined list (e.g., “It takes too long to get an appointment,” “I do not have insurance”), with instructions to check all that applied.

### 2.3 Data preparation

Prior to analysis, we addressed missing data, multicollinearity, and class imbalance using Python (version 3.12.4) and relevant libraries, including *pandas*, *numpy*, and *statsmodels*.

**Figure 1:**
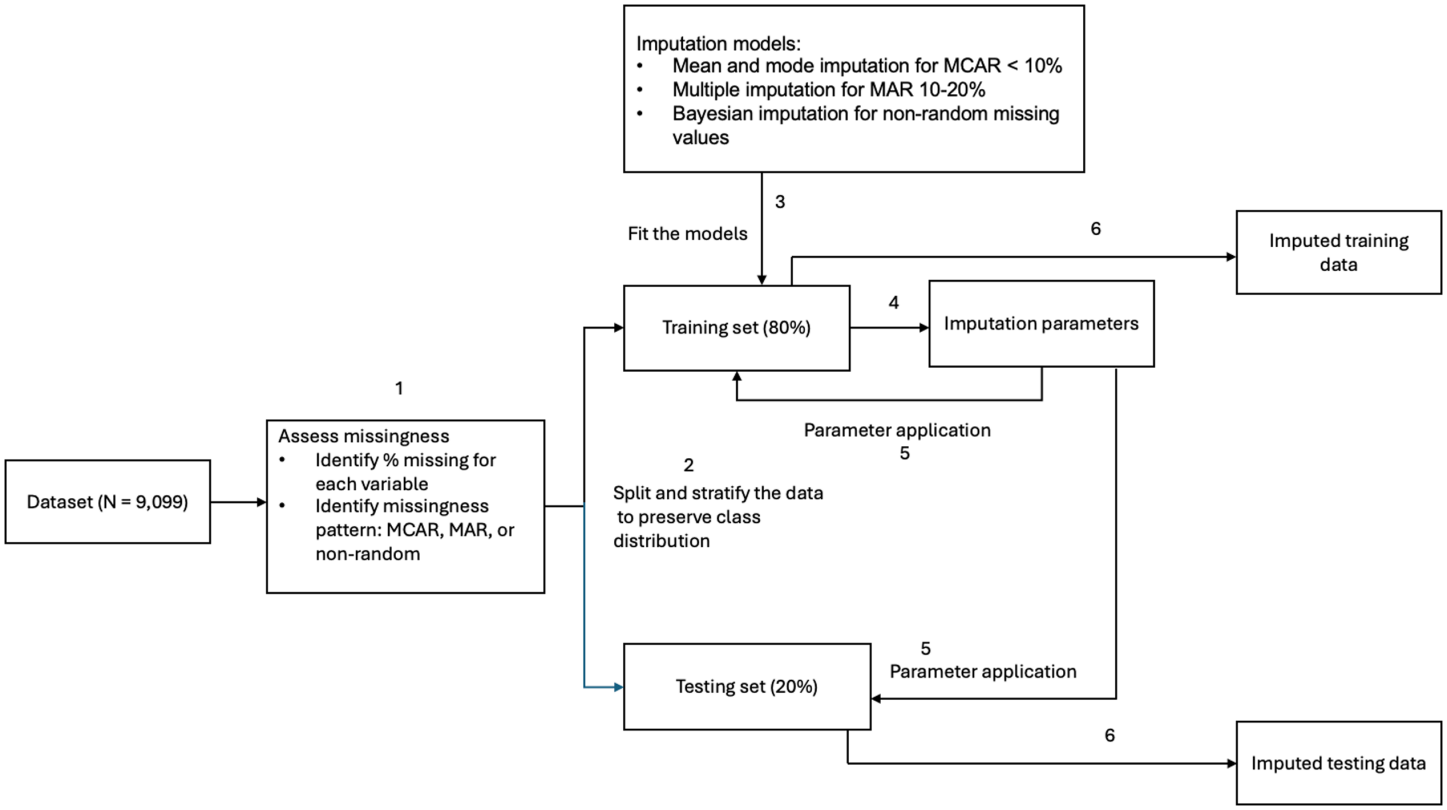
Missing data handling workflow.

For missing data, we first examined the underlying mechanisms. To avoid data leakage, all imputation procedures were performed after the initial train–test split, with imputation models fitted on the training data and applied to the test set. For predictive features missing completely at random and with less than 10% missingness, we applied mean imputation for continuous variables and mode imputation for categorical variables. For features missing at random (MAR) with 10–20% missingness, we used multiple imputation methods, supported by literature demonstrating their effectiveness for MAR data (30). Two predictive features—number of children under age 5 and number of children aged 5–18—exhibited nonrandom missingness at higher levels (20–35%). These were imputed using Bayesian modeling, which appropriately accounts for uncertainty in both the missingness mechanism and the imputation process (31). For the outcome variables, all healthcare needs and barriers to care domains had less than 5% missing data and were imputed using mode substitution. See Figure 1 for missing data handling workflow with train–test split before imputation to prevent data leakage.

Continuous predictors were not standardized, as most were measured on 5- or 6-point Likert-type scales. Standardization was not applied to preserve interpretability of model outputs. Multicollinearity was assessed using the variance inflation factor (VIF). All predictors demonstrated acceptable multicollinearity (VIF < 5) (32), with the exception of four county-level indicators, which had VIF values ranging from 5.7 to 7.8. These were retained due to their theoretical relevance and interpretive value. Retaining these indicators ensures that all counties remain represented in the models and preserves the utility of our findings for guiding county-specific interventions. Furthermore, the machine learning algorithms employed in this study (see Machine Learning Process) are generally robust to multicollinearity (33–35). While algorithms such as logistic regression and K-Nearest Neighbors are more sensitive to multicollinearity, the observed VIF levels remained within acceptable bounds (24, 30, 31).

To prevent data leakage and ensure valid model evaluation, all preprocessing involving categorical variables was performed after the initial train-test split (see Machine Learning Process). County indicators were encoded using one-hot encoding, as no single county should serve as a reference group given their equal importance, while binary variables such as gender were processed using label encoding, with male as the reference level in alignment with prior literature (17).

### 2.4 Model development

To address the complexity of relationships between sociodemographic, geographic, and household predictors and healthcare outcomes, a range of supervised machine learning approaches was employed. We implemented the models using Python (version 3.12.4) and leveraged libraries such as *scikit-learn, XGBoost, PyTorch*, and *imbalanced-learn*. We selected a diverse set of algorithms to capture linear, nonlinear, and multidimensional relationships in the data, reflecting varying mathematical assumptions and levels of representational flexibility. Seven algorithms spanning four modeling paradigms were tested: linear models (Logistic Regression, LASSO), tree-based models (Random Forest, Gradient Boosted Trees, and Adaptive Boosting), instance-based models (K-Nearest Neighbors), and deep learning models (Feedforward Neural Networks).

We selected a diverse set of predictive approaches to capture different ways of modeling relationships between predictors and healthcare outcomes. Linear models were included as interpretable baselines that assume additive, straight-line relationships. Tree-based models can automatically detect interactions and nonlinear patterns without extensive manual feature engineering. Instance-based methods handle more complex boundaries between outcome classes. Feedforward neural networks, although not strictly necessary for small predictor sets, were included to test whether their flexibility in learning subtle, nonlinear relationships and interactions could offer performance advantages in our context.

The dataset was split into 80% training and 20% testing subsets using stratified random sampling to preserve the distribution of outcome classes across both sets. For outcomes with class imbalance (e.g., mental healthcare needs; see Descriptive Statistics), we tested models both with and without class imbalance adjustment techniques. These included the Synthetic Minority Oversampling Technique, random oversampling of minority class instances, and cost-sensitive learning to adjust misclassification penalties. The version that yielded the best performance was selected for further evaluation. For outcomes with class balance (e.g., mental healthcare barriers; see Descriptive Statistics) or when the class of interest predominated (i.e., individuals reporting needs or barriers), we did not apply imbalance adjustments to avoid overfitting and preserve generalizability.

Model training included hyperparameter tuning and evaluation via five-fold cross-validation on the training set. Hyperparameters were optimized using both grid search and randomized search to identify the best-performing configuration for each algorithm. After optimization, we evaluated the final models on the unseen testing set to estimate out-of-sample performance. For Random Forest models, we also employed out-of-bag error estimation as an additional validation method. This approach uses bootstrapped samples during training, leaving about one-third of the data unused by individual trees. These out-of-bag samples served as a pseudo-validation set, offering an unbiased estimate of performance during training.

### 2.5 Evaluation metrics

Performance was assessed using recall (the proportion of true positives correctly identified), precision (the proportion of predicted positives that are true positives), F1-score (the harmonic mean of precision and recall), accuracy (the proportion of all correct predictions), and specificity (the proportion of true negatives correctly identified). In alignment with public health priorities, we emphasized maximizing recall to reduce false negatives and ensure that individuals with healthcare needs or access barriers were not overlooked. For each outcome, we selected the best-performing algorithm based on recall and used the area under the precision-recall curve (AUC-PR) to evaluate whether adjusting classification thresholds could further enhance recall while maintaining acceptable precision. A precision range of approximately 30–50% was deemed acceptable in order to maximize identification of high-need individuals, consistent with public health approaches that prioritize high recall despite a moderate increase in false positive (36).

Interpretability was a key focus throughout the modeling process. For linear models, feature importance was assessed using the magnitude of the model coefficients. For tree-based models, feature importance was evaluated using both Gini importance and permutation importance. For kernel-based and instance-based models, permutation importance was applied to assess feature relevance by observing the impact on model performance when features were shuffled. For all models mentioned, Partial Dependence Plots (PDPs) were generated to examine the marginal effect of each predictor on the outcome while holding all other variables constant. PDPs provide valuable insights into the direction and strength of relationships between predictors and the outcome. For example, they can illustrate whether increasing a variable like family support is associated with a higher or lower predicted probability of reporting a dental healthcare barrier.

**Figure 2:**
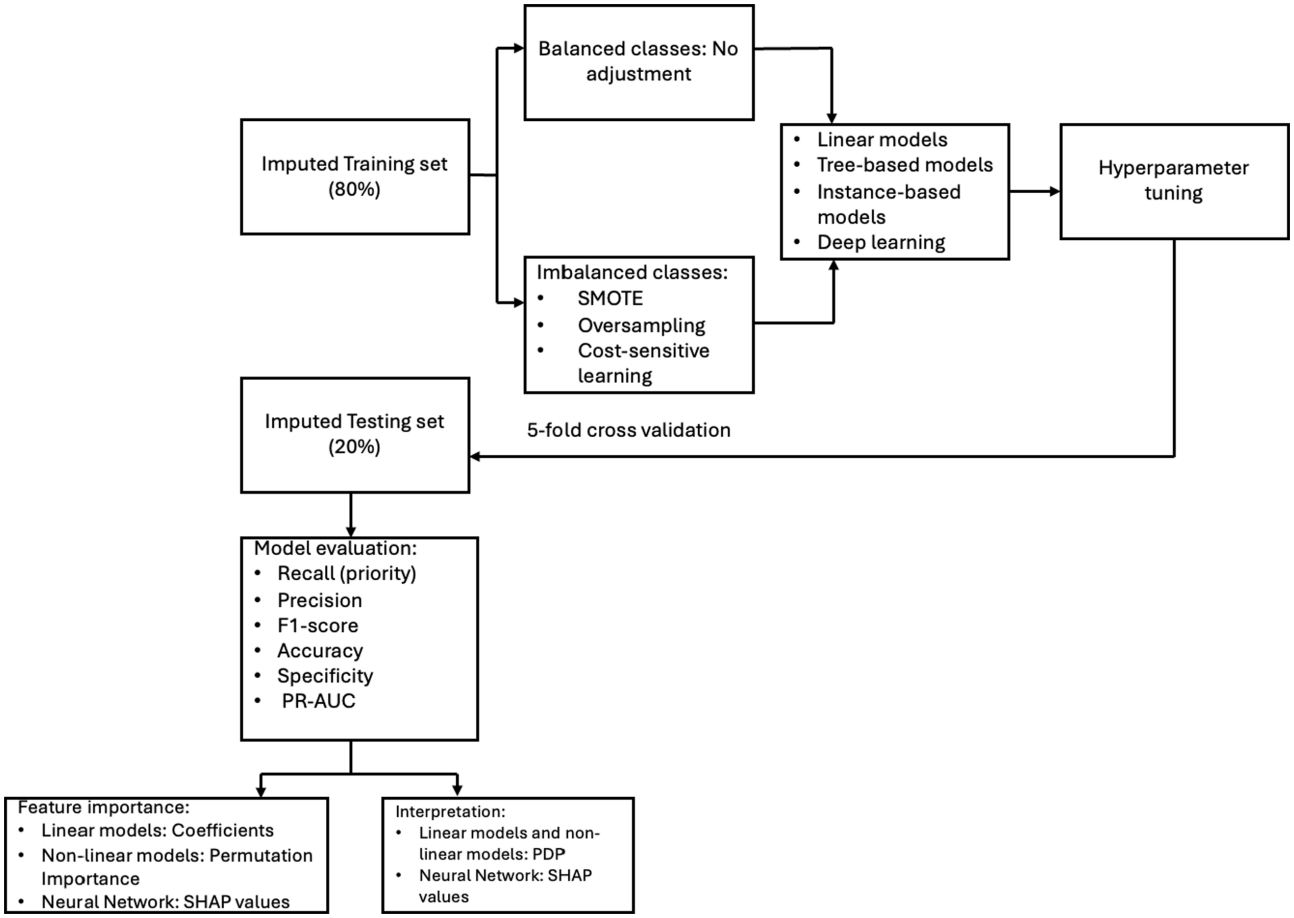
Model development and evaluation framework.

For feedforward neural networks, we used SHapley Additive exPlanations (SHAP) to assess both feature importance and the direction of effect. SHAP values estimate the contribution of each feature to the model’s prediction for each individual. Positive SHAP values indicate that a feature increases the predicted probability of the outcome, while negative values indicate a decrease. SHAP summary plots also include a color gradient (see Results)—ranging from blue (low feature values) to red (high feature values) in our study—which helps interpret how the magnitude of a feature relates to its impact. For example, red points clustered on the positive side of the SHAP axis indicate that high values of that feature increase the likelihood of the outcome. Dots that are tightly clustered suggest a consistent influence of the feature across individuals, while widely dispersed dots reflect more variability in the feature’s impact on predictions. See Figure 2 for the model development pipeline and evaluation framework.

## 3 Findings

### 3.1 Descriptive statistics

Table 1 summarizes the demographic, household, and label distributions of the analytic sample (*N* = 9,099). The sample included a broad age range with the largest proportion aged 55 and older, and the majority of respondents identified as female and Hispanic or White. Smaller proportions identified as Native American, Black, or Asian, reflecting the population distribution of New Mexico counties. The average reported household income fell within the $40,000–$54,999 range (*M* = 3.90, *SD* = 1.75, based on ordinal income coding).

**Table 1:** Sample characteristics of survey respondents (*N* = 9,099).

Variable	*n*	%
Age		
18–24	400	4.4
25–34	1,529	16.8
35–44	1,265	13.9
45–54	1,656	18.2
55+	2,202	24.2
Gender		
Female	6,333	69.6
Male	1,929	21.2
Race/Ethnicity		
Hispanic	3,867	42.5
White	3,494	38.4
Native American	555	6.1
Black	164	1.8
Asian	91	1.0
Household size		
1	801	8.8
2	1,677	18.4
3	1,468	16.1
4	1,587	17.4
5	836	9.2
6	323	3.6
7+	195	2.1
Children in household		
Responsible for children <18 (Yes)	5,277	58.0
Responsible for children <18 (No)	3,712	40.8
Number of children <5 = 0	3,607	39.6
1	1,493	16.4
2	632	6.9
3	152	1.7
4+	92	1.0
Number of children 5–18 = 0	1,386	15.2
1	2,041	22.4
2	1,609	17.7
3	617	6.8
4+	332	3.6
Household income		
Less than $10,000	796	8.7
$10,000–24,999	1,212	13.3
$25,000–39,999	1,259	13.8
$40,000–54,999	1,091	12.0
$55,000–69,999	928	10.2
$70,000+	2,212	24.3
County of residence		
Doña Ana	1,224	13.5
Santa Fe	1,071	11.8
Curry–Roosevelt	1,070	11.8
Socorro	952	10.5
Bernalillo	878	9.7
Valencia	838	9.2
San Juan	778	8.6
Taos	546	6.0
Otero	474	5.2
Rio Arriba	424	4.7
San Miguel	369	4.1
Guadalupe	277	3.0
Catron	198	2.2
Perceived family support		
Strongly disagree (1)	1,581	17.4
Disagree (2)	962	10.6
Neither agree nor disagree (3)	1,220	13.4
Agree (4)	2,484	27.3
Strongly agree (5)	2,003	22.0
Healthcare needs (labels)		
Dental need (Yes)	7,643	84.0
Dental need (No)	1,456	16.0
Medical need (Yes)	8,007	88.0
Medical need (No)	1,092	12.0
Mental health need (Yes)	3,549	39.0
Mental health need (No)	5,550	61.0
Healthcare barriers (labels)		
Dental barrier (Yes)	2,548	28.0
Dental barrier (No)	6,551	72.0
Medical barrier (Yes)	3,731	41.0
Medical barrier (No)	5,368	59.0
Mental health barrier (Yes)	4,550	50.0
Mental health barrier (No)	4,550	50.0

For certain variables, counts do not sum to *N* = 9,099 due to missing data. Percentages are calculated using the full sample as the denominator.

Over half of respondents reported responsibility for children under 18, and average household size was just over three people (*M* = 3.25, *SD* = 1.5). For the number of children aged 5–18, the average number was (*M* = 1.44; *SD* = 1.18). For the number of children under the age of five, the average number was 0.60 (*SD* = 0.89). The three counties with the highest representation were Doña Ana (13.5%), Santa Fe (11.8%), and the combined counties of Curry and Roosevelt (11.8%). Perceived family support, measured on a 5-point Likert scale, had a mean score of 3.29 (*SD* = 1.44), corresponding roughly to “neither agree nor disagree.”

Label distributions highlight the imbalance across domains: needs were most frequently reported in the medical (88%) and dental (84%) domains, while mental health needs were less common (39%). In contrast, barriers were most frequently reported for mental health (50%) and medical care (41%), compared with dental care (28%). These patterns indicate that although perceived needs were widespread across medical and dental care, perceived barriers were more concentrated in mental health, underscoring the importance of accounting for class imbalance in subsequent modeling.

### 3.2 Machine learning results

#### 3.2.1 Mental health care needs

Random forest with balanced class weights demonstrated strong performance in predicting mental health care needs, achieving a recall of 0.97, precision of 0.40, F1-score of 0.75, and accuracy of 0.42 at a decision threshold of 0.40. The specificity was 0.84. The area under the precision-recall curve (PR-AUC), calculated across all thresholds, was 0.49. The top five predictors included age, family support, income, household size, and gender (see Supplementary Table 1). Specifically, younger individuals were more likely to be predicted as needing mental health support, with predicted probability decreasing significantly in the oldest age group (age 55+).

**Figure 3:**
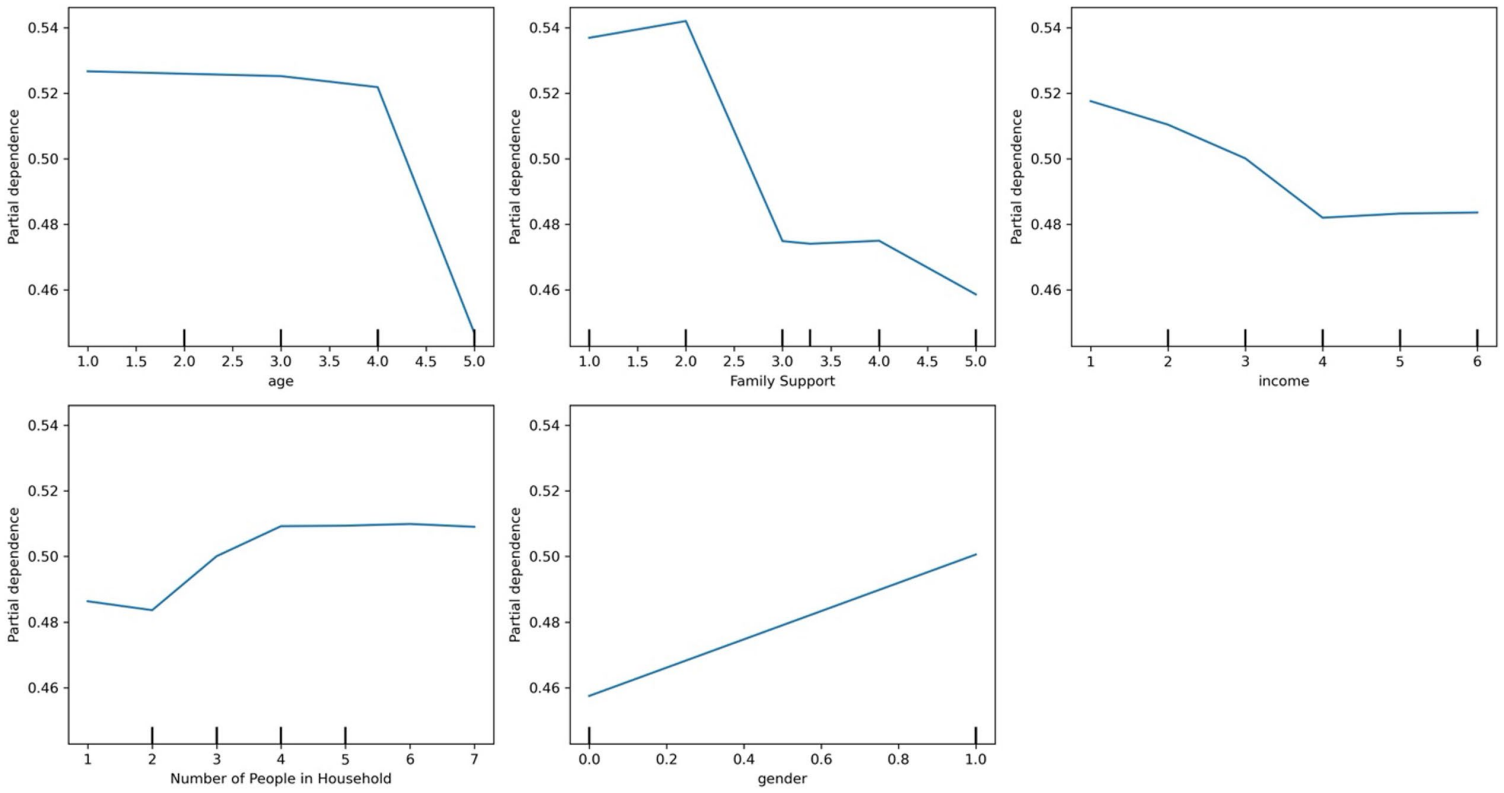
Partial dependence plots of the top predictors for mental health care needs using random forest.

As shown in Figure 3, which presents a partial dependence plot for the top predictors, perceived family support had a strong inverse relationship with predicted need—individuals reporting lower support were more likely to be classified as needing care. Similarly, lower income levels were associated with higher predicted need, although the effect plateaued at higher income brackets. Larger household size slightly increased the predicted probability of needing mental health services. Finally, women were more likely than men to be predicted as needing mental health support.

#### 3.2.2 Medical care needs

**Figure 4:**
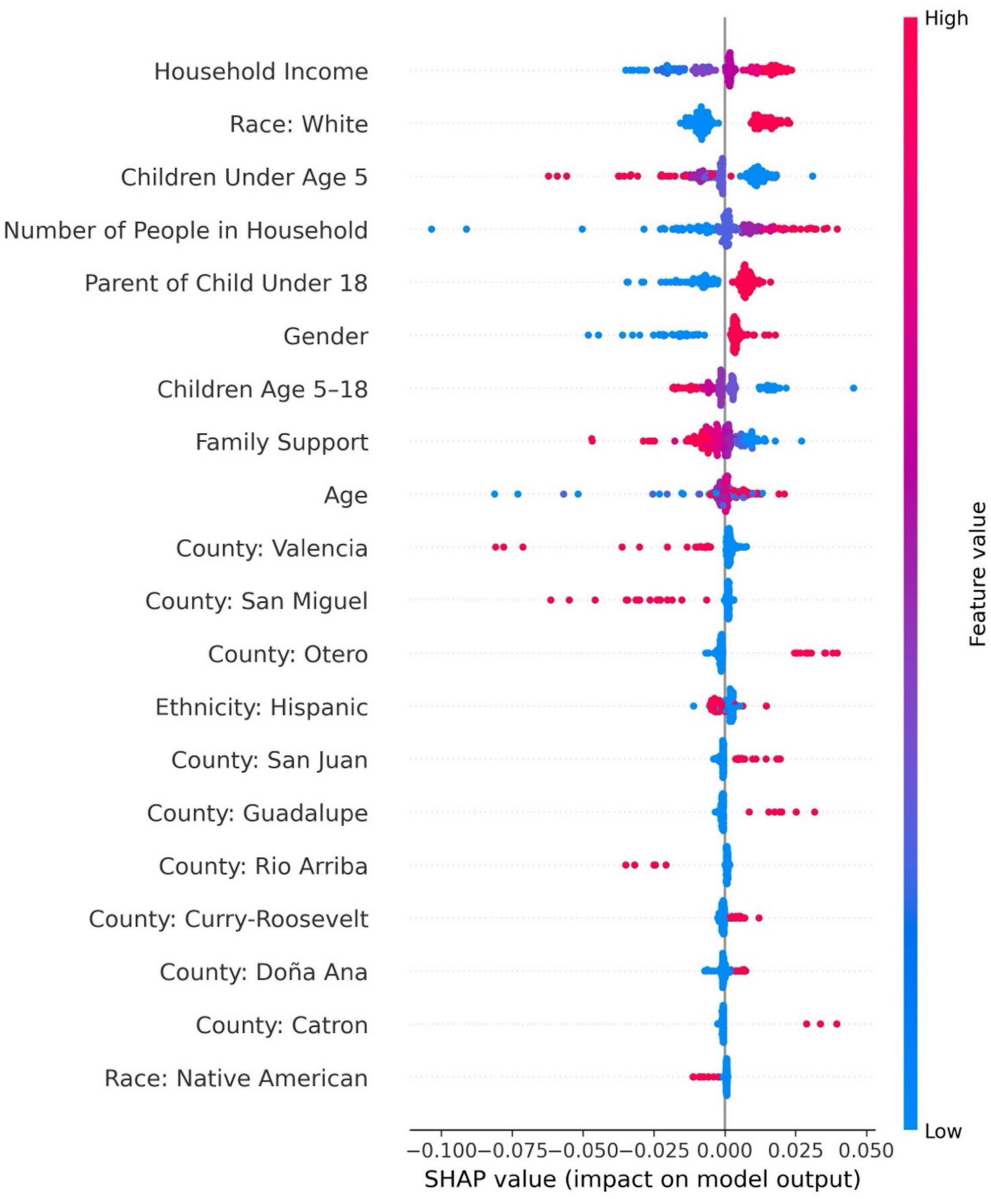
SHAP Summary Plot for Predicting Medical Care Needs Using a Feedforward Neural Network.

The feedforward neural network was the best-performing model for predicting medical care needs, achieving a recall of 0.99, an F1-score of 0.94, precision of 0.90, accuracy of 90%, specificity of 0.99 at a decision threshold of 0.5. The area under the PR-AUC was 0.93. The top five most influential features included income, race, household size, number of children under five, and parental status (see Supplementary Table 2). As shown in Figure 4, the SHAP summary plot depicts the ranked importance of features and their direction of effect, showing that having more children under age five was associated with a lower likelihood of needing medical care, which may reflect that caregivers with multiple children are more likely to delay or deprioritize their own healthcare due to competing demands. In contrast, being a parent overall (regardless of the number of children) was associated with greater predicted need, possibly indicating heightened awareness of health issues that accompany parenting responsibilities. Identifying as White, having higher income, and living in a larger household were also associated with increased medical care needs. SHAP values for household size were widely dispersed, suggesting heterogeneity in its effects across individuals—likely reflecting interaction with other sociodemographic or contextual factors.

#### 3.2.3 Dental care needs

**Figure 5:**
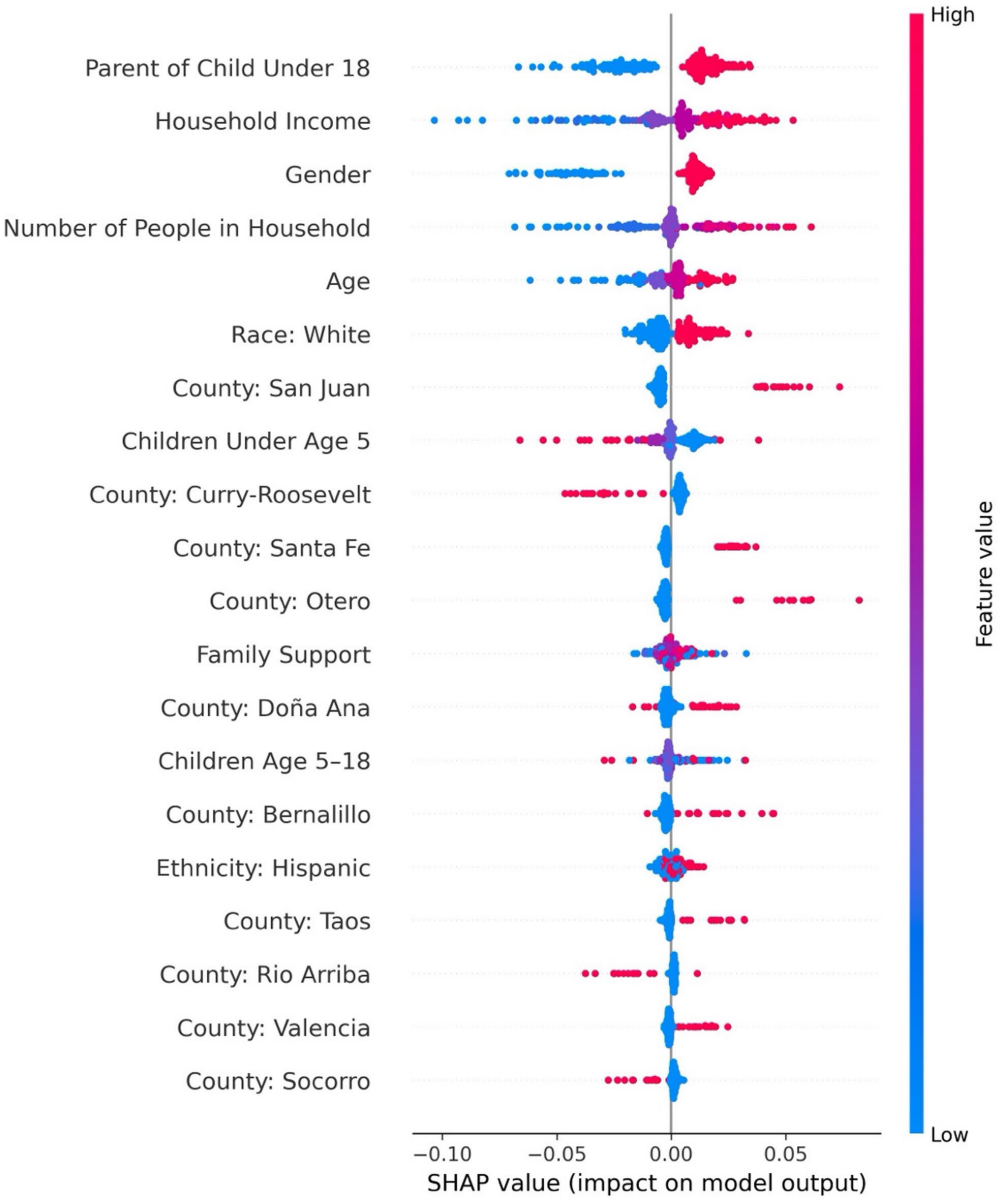
SHAP summary plot for predicting dental care needs using a feedforward neural network.

The feedforward neural network demonstrated the highest performance in predicting dental care needs, achieving a recall of 0.99, precision of 0.87, F1-score of 0.93, accuracy of 87%, and specificity of 0.98 at a decision threshold of 0.40. The area under the precision-recall curve (PR-AUC), calculated across all thresholds, was 0.94. The most influential predictors included parental status, income, gender, age, and race (see Supplementary Table 3). Figure 5’s SHAP summary plot illustrates these relationships, showing that being a parent, having a higher income, identifying as White, being female, and older age were associated with an increased likelihood of needing dental care. In contrast, although it was not among the top five features, having fewer young children under age five in the household was associated with greater dental care needs. This pattern may reflect that adults in households with fewer young children have more capacity to recognize and attend to their own dental care needs. Notably, the SHAP values for race showed that individuals who did not identify as White were tightly clustered, suggesting a consistent pattern of lower dental care needs among those groups.

#### 3.2.4 Mental health care barriers

**Figure 6:**
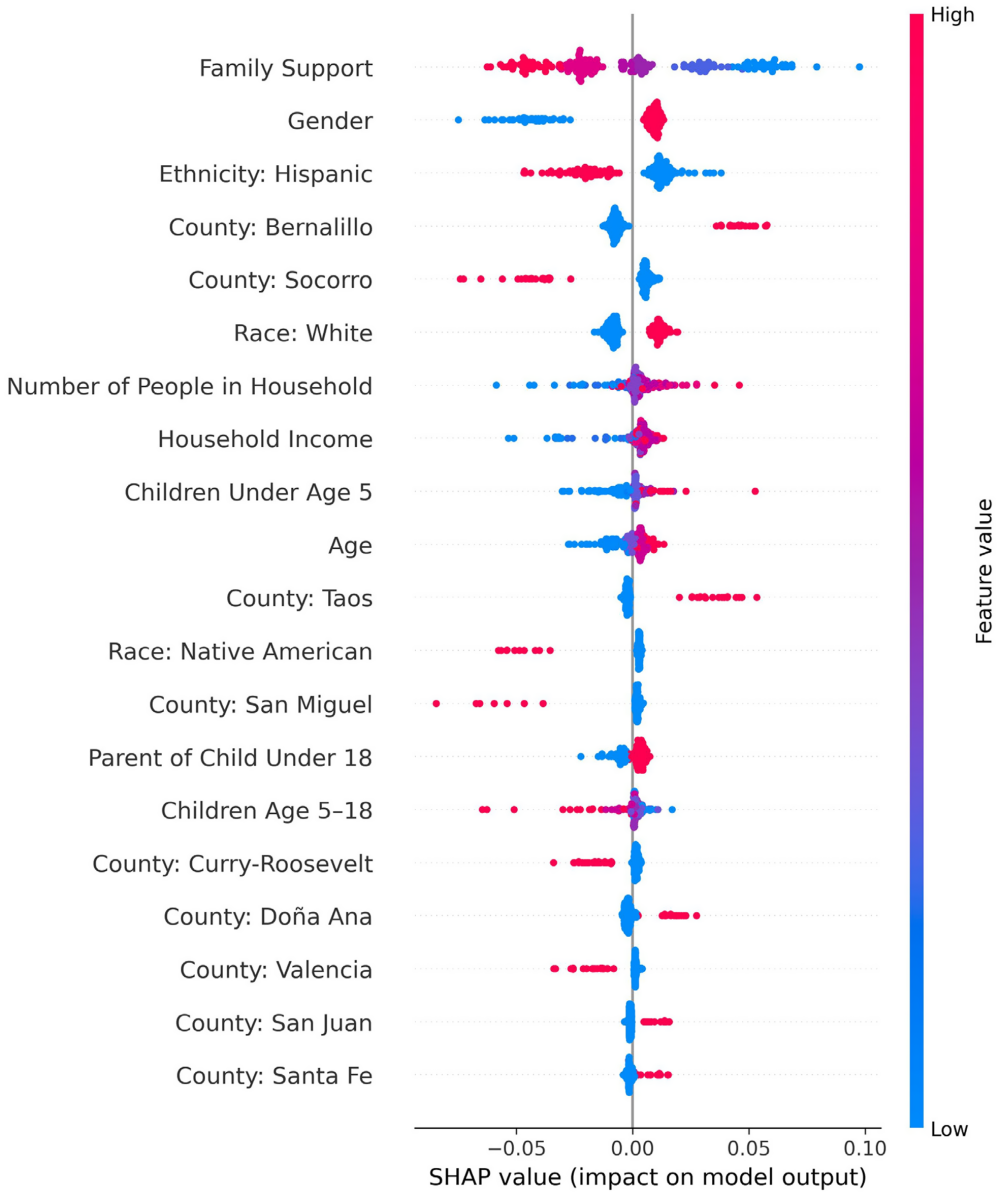
SHAP summary plot for predicting mental healthcare barriers using a feedforward neural network.

A feedforward neural network was the best-performing model for predicting mental health care barriers, achieving a recall of 0.94, precision of 0.52, F1-score of 0.67, accuracy of 53%, and specificity of 0.90 at a decision threshold of 0.5. The precision-recall area under the curve (PR-AUC), calculated across all thresholds, was 0.56. The top five predictors included family support, race, gender, urban residence, and rural residence (see Supplementary Table 4). As shown in Figure 6, the SHAP summary plot reveals both the magnitude and direction of feature effects, with lower levels of family support, being female, and residing in large metro counties such as Bernalillo were associated with greater mental health care barriers. In contrast, identifying as Hispanic and living in smaller rural counties such as Socorro predicted fewer barriers. Notably, the SHAP values for family support and Bernalillo County were widely dispersed, suggesting substantial variability in how these factors influence access. In contrast, SHAP values for individuals residing outside Bernalillo County were tightly clustered, indicating more consistent associations with lower barriers in those populations.

#### 3.2.5 Medical barriers

**Figure 7:**
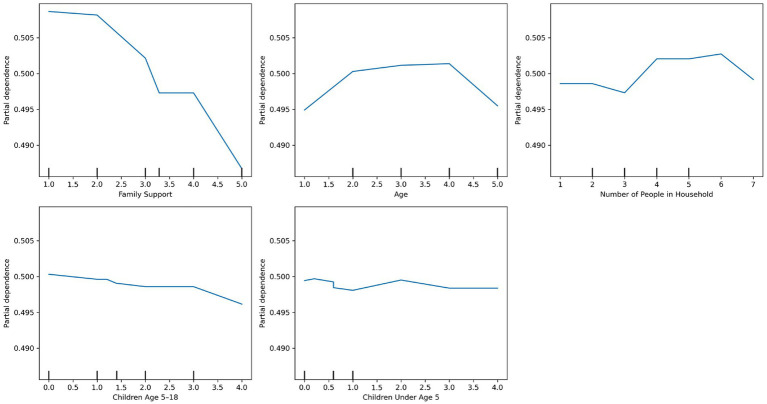
Partial dependence plots of the top predictors for medical care barriers using adaptive boosting.

The adaptive boosting model with class weight adjustment achieved the highest performance in predicting barriers to medical care, yielding a recall of 0.99, precision of 0.41, F1-score of 0.58, accuracy of 94%, and specificity of 0.84 at a decision threshold of 0.40. The area under the precision-recall curve (PR-AUC), calculated across all thresholds, was 0.51. The top five predictors were family support, age, household size, the number of children aged 5–18, and the number of children under five (see Supplementary Table 5). Figure 7 shows the partial dependence plot for these predictors, where lower levels of family support were strongly associated with increased barriers to care. Age and household size showed modest, nonlinear effects: barriers peaked among middle-aged individuals (45–54) and in moderately sized households (four members), but declined among older adults and larger households. The number of school-aged children had minimal influence, while having more children under five slightly reduced barriers—potentially due to increased eligibility for targeted services. Overall, family support emerged as the most influential factor in reducing medical care barriers.

#### 3.2.6 Dental barriers

**Figure 8:**
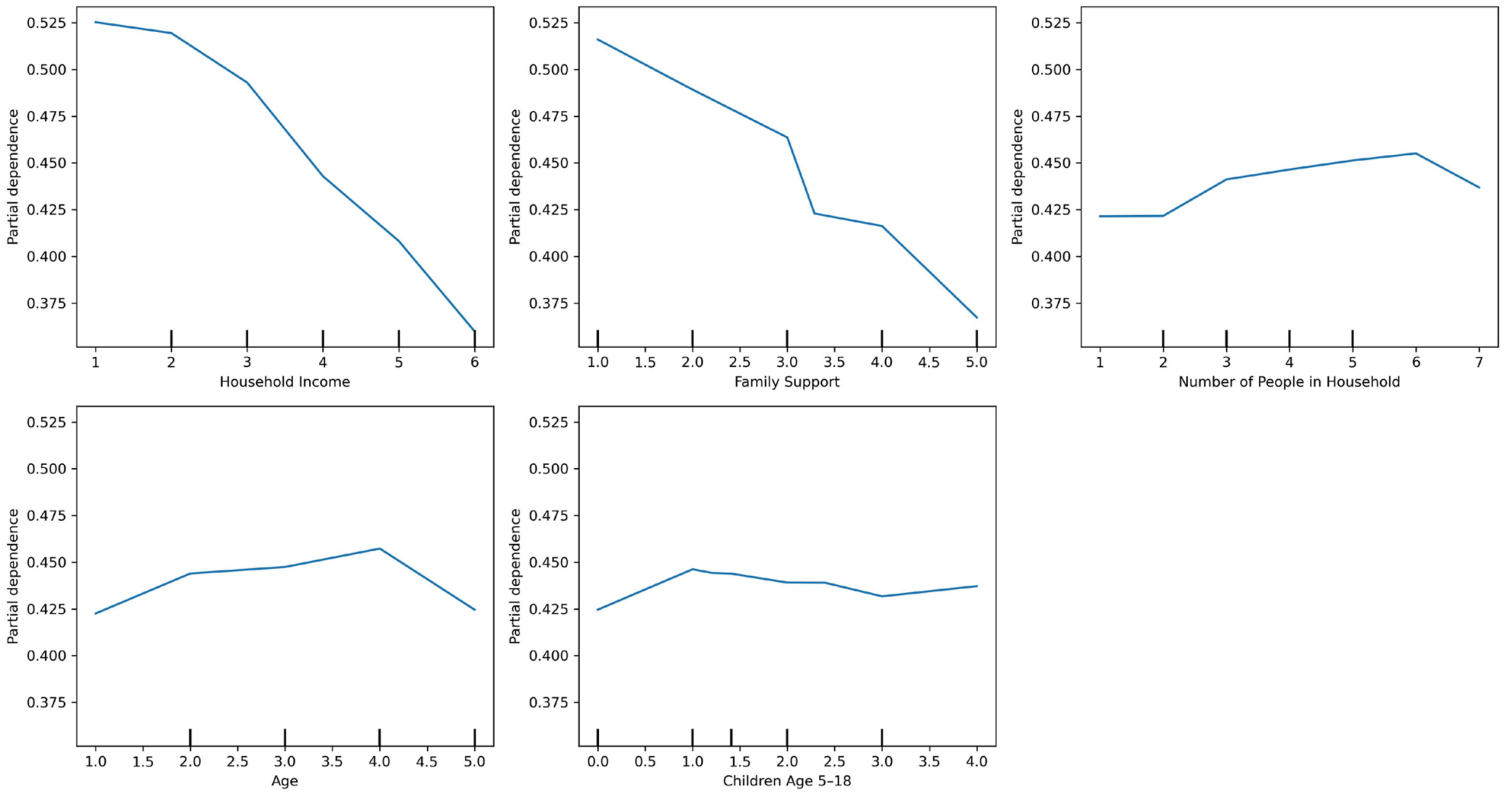
Partial dependence plots of the top predictors for dental care barriers using random forest.

The random forest model with balanced class weights outperformed other models in predicting dental care barriers, achieving a recall of 0.98, precision of 0.30, F1-score of 0.45, accuracy of 34%, and specificity of 0.75 at a decision threshold of 0.40. The area under the precision-recall curve (PR-AUC), calculated across all thresholds, was 0.41. The top five predictors included family support, income, household size, age, and the number of children aged 5–18 (see Supplementary Table 6). As shown in Figure 8, which presents a partial dependence plot for these features, higher perceived family support and income were associated with lower barriers. Barriers peaked among middle-aged adults (45–54), then declined slightly among those aged 55 and older, although the effect of age was relatively modest.

### 3.3 Barriers to access distribution

For people selecting yes to barriers to care, they checked the list of barriers for each domain as present in Table 2.

**Table 2:** Count and percentage distribution of reported barriers to dental, medical, and mental health care.

Barrier	Dental, *n* (%)	Medical, *n* (%)	Mental health, *n* (%)
Takes too long to get care	1,197 (13.15%)	1,684 (18.51%)	1,345 (14.78%)
Costs too much	1,434 (15.76%)	893 (9.81%)	803 (8.83%)
Cannot find a quality provider	705 (7.75%)	937 (10.30%)	1,173 (12.89%)
Appointment times do not work	613 (6.74%)	660 (7.25%)	709 (7.79%)
Cannot find a specialist	337 (3.70%)	756 (8.31%)	550 (6.04%)
Cannot find a provider accepting new patients	368 (4.04%)	605 (6.65%)	775 (8.52%)
Does not accept insurance	563 (6.19%)	532 (5.85%)	535 (5.88%)
No insurance	806 (8.86%)	430 (4.73%)	366 (4.02%)
Appointments canceled due to COVID-19	430 (4.73%)	582 (6.40%)	389 (4.27%)
Too far to travel	483 (5.31%)	449 (4.93%)	399 (4.39%)
High co-pays	480 (5.28%)	399 (4.38%)	318 (3.50%)
Do not know where to get care	288 (3.16%)	259 (2.85%)	619 (6.80%)
Not enough insurance	516 (5.67%)	221 (2.43%)	210 (2.31%)
No transportation	304 (3.34%)	257 (2.82%)	211 (2.32%)
Worried/afraid about going	262 (2.88%)	198 (2.18%)	298 (3.28%)
Feel bad about going	211 (2.32%)	167 (1.83%)	308 (3.38%)
Language barrier	102 (1.12%)	69 (0.76%)	92 (1.01%)

Percentages reflect the proportion of total respondents (*N* = 9,099) selecting each barrier. Because participants could select more than one barrier within each healthcare domain, counts and percentages do not sum to 100%.

The ranked table above illustrates the most frequently reported barriers across healthcare domains. Consistently across dental, medical, and mental health services, the most common obstacles were related to delays in care, cost, and difficulty finding quality providers. These system-level barriers were closely followed by issues with appointment scheduling and limited availability of accepting providers. Psychosocial factors such as fear or stigma were more salient in the mental health domain than in others, while logistical issues like transportation and language barriers were reported less frequently across all domains. Collectively, these patterns underscore the complex interplay between structural, logistical, and emotional barriers in accessing care—especially within underserved populations in high-disparity settings like New Mexico. Full performance metrics for all tested models across each outcome are provided in Supplementary Table 7.

## 4 Discussion

Healthcare needs and access barriers across mental, medical, and dental domains are shaped by distinct patterns of sociodemographic, geographic, and household factors, underscoring the importance of domain-specific, data-driven interventions. By applying machine learning (ML) models that accommodate nonlinear and interacting effects, we identified nuanced predictors of both need and access across the healthcare continuum. These models offer a more flexible and context-sensitive alternative to traditional statistical methods and help uncover hidden disparities in complex social systems (25, 26).

This study addressed four primary aims. Aim 1 was met by identifying features—such as household size, income, parental status, and family support—that most strongly predicted perceived healthcare needs and access barriers. Aim 2 involved comparing ML model performance across medical, dental, and mental health domains; our results showed that nonlinear models, particularly neural networks, offered improved prediction accuracy and granularity over linear counterparts. Aim 3 was addressed through descriptive analyses of self-reported reasons for barriers to care, which revealed that cost, long wait times, and difficulty finding quality providers were the most commonly cited obstacles. Aim 4 was fulfilled by using PDP and SHAP values to generate interpretable insights at both individual and population levels, offering actionable knowledge for targeted interventions.

### 4.1 Aim 1: predictors of perceived needs and barriers

#### 4.1.1 Mental health: converging needs and barriers

Mental health care showed the clearest overlap between predictors of need and access barriers. Consistent with prior research, younger adults, women, individuals with lower income, and those with limited family support were more likely to report mental health care needs (37, 38). Importantly, women and residents of urban counties like Bernalillo were also more likely to face barriers to accessing that care, indicating a compounding vulnerability—where those most in need of services also encounter the most difficulty obtaining them. Different factors are related to need and access between rural and urban areas; in urban areas, there are more providers, higher rates of diagnoses among youth in comparison with their rural counterparts, and access barriers related to cost and significantly higher rates of providers not accepting insurance compared with non-urban areas (39–41). Rurality is related to fewer providers and geographic barriers (42). In New Mexico, cultural taboos and stigma influence seeking mental health care, especially in urban areas (43), demonstrating that both structural and contextual dynamics influence access within the state (9, 10).

Interestingly, identifying as Hispanic predicted lower reported barriers to mental health care. Existing research has found Hispanic adults are more likely to report barriers to care compared to their White counterparts (23). Our results may relate to strong family or community-based support systems among Hispanics in our study or foregoing care, rather than ease of access to formal mental health care services (44). Results may also be due to our analytic approach. Our model used one-hot encoding with “non-Hispanic” as the reference group, encompassing all racial and ethnic identities not identifying as Hispanic (e.g., White, Black, Asian). In contrast, many earlier studies compared Hispanic respondents specifically to non-Hispanic White persons (23). Thus, the negative association observed here may reflect fewer perceived barriers relative to a broader, more diverse non-Hispanic group. It is also possible that structural barriers like cost or insurance remain, but cultural norms around family and community support help buffer perceived limitations—especially in regions like New Mexico with large, long-established Hispanic populations.

#### 4.1.2 Dental care: diverging needs and barriers

In contrast, the dental care domain demonstrated a sharp divergence between who needs care and who struggles to access it. Predicted dental needs were highest among White women who were parents, lived in smaller households, and had higher incomes—groups that may be more aware of dental care norms and more likely to seek preventive treatment. However, those facing the greatest barriers were typically older, low-income individuals with limited family support. This divergence likely reflects differences in both awareness and access. Higher-income White parents, who were predicted to have the greatest dental care needs, may have greater awareness of oral health norms and a stronger tendency to seek preventive or elective care, which increases self-reported need. In contrast, the groups facing the highest barriers—older adults, low-income individuals, and those with limited family support—may experience fewer self-reported needs due to lower utilization, reduced expectations of care, or adaptation to chronic oral health conditions.

The findings align with previous studies (14) indicating large differences in seeking dental care by income, with over twice the rates of receiving dental care and preventive visits among higher compared with lower income individuals. The difference between perceived needs (White mothers with higher incomes) and barriers (older, low income) in our findings suggests that in dental care, perceived or reported need equates to access while reporting lesser need relates to more systemic barriers.

#### 4.1.3 Medical care: contextual complexity

Patterns of medical care needs and barriers revealed nuanced, nonlinear relationships that highlight the complexity of healthcare access in diverse, resource-limited settings like New Mexico. Aligned with previous research (19, 20), access barriers to medical care were more likely among individuals with larger household sizes, those in midlife (particularly ages 45–54), and those with limited family support. These factors likely represent a combination of emotional and logistical strain—balancing caregiving, aging, and healthcare needs with limited social or structural resources.

Notably, parental status and larger household size were associated with greater predicted medical care needs, likely reflecting increased caregiving responsibilities and the cumulative health demands within multi-person households. However, having a greater number of children—particularly those under age 5 or between ages 5–18—was associated with lower predicted needs. This suggests that parent status alone is related to perceived health, or that these could also be multi-generational or multi-family households. Previous research links parenting to physical and emotional stress; decreased capacity for self-care such as physical activity; and higher rates of some health conditions such as obesity (45). Families with more children may span age-ranges that afford access to preventive care systems such as school-based health services or early childhood programs (45). It is also possible that these parents deprioritize their own care or underreport their needs while focusing on their children’s health. Thus, being a parent increases overall medical need, but the number of children may act as a protective factor through increased system contact or shifts in self-reporting behavior.

Interestingly, White individuals were more likely to be predicted as needing medical care, which may reflect greater healthcare utilization or health-seeking behavior rather than a higher actual burden of illness. This aligns with prior research showing that Hispanic, Native American, and other racially minoritized groups often face systemic barriers to care (9, 10), which may result in the underdiagnosis or underreporting of medical needs, even when the risk is elevated.

### 4.2 Aim 2: comparing model performance

Model performance varied by outcome domain, reflecting differences in the underlying data patterns for needs versus barriers. Neural networks consistently achieved the highest recall and overall performance in predicting healthcare needs—particularly for medical and dental care—likely due to their capacity to capture complex nonlinear interactions among sociodemographic and household factors. In contrast, tree-based ensemble methods such as random forest and adaptive boosting often outperformed neural networks for barriers outcomes, suggesting that barriers may be driven by a more limited set of influential features with hierarchical interactions that these models can detect efficiently. Linear models, while offering interpretability, generally underperformed relative to nonlinear approaches, underscoring the value of flexible algorithms when modeling high-dimensional social determinant data. These differences have practical implications for public health applications: model choice should be guided not only by predictive accuracy but also by the type of outcome (need versus barrier), the complexity of relationships among predictors, and the balance between interpretability and performance for stakeholder decision-making.

### 4.3 Aim 3: reasons for barriers to care

Across all domains, family support emerged as a strong, consistent protective factor against barriers to care, aligning with prior research emphasizing the role of social support in mitigating access challenges (19, 20). Race and geography also played substantial roles: White individuals were more likely to be predicted as needing care—possibly reflecting greater healthcare engagement—while Hispanic and Native American individuals may have underreported need due to systemic underdiagnosis or limited access. While rurality typically increased barriers, some smaller rural counties exhibited lower predicted barriers, suggesting that strong community infrastructure, cultural cohesion, or targeted local programs may mitigate broader structural disadvantages.

Importantly, descriptive analyses of self-reported reasons for access barriers provided further context for these patterns. Consistently across dental, medical, and mental health domains, the most common barriers included long wait times, high costs, and challenges finding quality providers. These structural obstacles echo previous national findings on cost and availability as key access deterrents (20, 22). Scheduling difficulties and limited acceptance of new patients also ranked high, while psychosocial barriers—such as fear and stigma—were more salient in the mental health domain. In contrast, logistical factors like transportation or language barriers were reported less frequently. These trends suggest that system-level reform may be more urgently needed than individual-level behavior change, particularly in underserved areas where the burden of navigating care often falls disproportionately on low-income or minoritized individuals (9, 19, 23).

### 4.4 Aim 4: policy implications

The findings from this study offer several important implications for health equity efforts in New Mexico and similar high-disparity, rural settings. First, the divergence between groups with high healthcare needs and those facing the most significant barriers highlights the necessity of distinguishing between need and access when designing interventions. In domains like dental care, interventions should not only target individuals with high utilization or perceived need (e.g., White, higher-income parents) but also prioritize those structurally excluded from care—particularly older adults and low-income individuals.

Second, the convergence of need and barriers in mental health—especially among women, younger adults, and those with low family support—calls for integrated and gender-sensitive mental health programs. This could include expanding mobile behavioral health services, peer navigation supports, school-based or integrated primary care-behavioral health, and culturally tailored community outreach in both urban and rural counties. The finding that Hispanic individuals face fewer barriers may reflect the protective role of informal community supports, suggesting that public health efforts should invest in community-based models and partnerships with trusted local organizations, especially in Hispanic and Native American communities. Counties in this study are involved in a comprehensive initiative to reduce adversity and address barriers to services among all New Mexicans—*100% Community*—demonstrating such a model. In Montana, similar in its predominantly rural landscape, over 60 primary care settings have integrated behavioral health, and case management has also been expanded through recent initiatives to facilitate mental health complexity (46).

Third, family support emerged as a key cross-cutting protective factor in reducing access barriers across all care domains. Policymakers should consider programs that strengthen informal caregiving networks—such as family caregiver stipends, flexible work arrangements for caregivers, or intergenerational support hubs. In a state like New Mexico, where geographic isolation and under-resourced formal systems are common, family-centered strategies may offer sustainable, culturally resonant solutions to healthcare inequities.

Fourth, the variation in barriers across counties—particularly the lower barriers in small rural counties like Socorro and San Miguel—suggests that county-specific investments and localized planning matter. Rather than relying on one-size-fits-all state-wide solutions, policymakers should prioritize hyperlocal planning and data-driven targeting to amplify what’s working in lower-barrier counties and address service gaps in higher-barrier ones like Taos and Bernalillo. New Mexico’s approach, collecting the local data in this study to inform county-specific solutions demonstrates data-driven targeting. Other efforts include local Community Health Improvement Plans and hyper-local planning is increasingly common in a number of socio-ecological initiatives (47).

Finally, this study demonstrates the utility of interpretable machine learning as a public health planning tool. By revealing complex, nonlinear relationships among predictors, ML can inform more precise risk stratification and resource allocation than traditional methods. Public health agencies should consider integrating ML models into routine health surveillance systems to monitor emerging needs and direct interventions where they are most needed, especially in resource-limited settings. The use of interpretable model outputs, such as SHAP values and partial dependence plots, can facilitate stakeholder engagement by making complex predictive findings more transparent and actionable. Public health, behavioral health, and other community planners could use a survey such as 100% Community in combination with interpretability analyses to prioritize and tailor outreach. This could include using a dashboard to depict the salience of each factor in very precise areas of their community. By visually showing how sociodemographic and household factors contribute to predicted needs or barriers, these tools can help local health departments, community coalitions, and service providers prioritize resources, build consensus, and design interventions aligned with local priorities.

### 4.5 Equity implications and fairness considerations

In line with human-centered computing principles, it is critical to interpret the findings of our machine learning models with attention to equity and fairness. Our sample composition—most participants identifying as Hispanic (42.5%) or White (38.4%), with smaller proportions identifying as Native American (6.1%), Black (1.8%), and Asian (1.0%)—reflects the demographic distribution of many New Mexico counties (9, 10). While class imbalance adjustment techniques were applied during model development, the underrepresentation of certain racial groups means that the models may have reduced predictive accuracy for these populations. Similarly, 69.6% of respondents identified as female, 21.2% as male, and 9.2% were coded as missing, including non-binary or “prefer not to answer” responses. Given the limited representation of non-binary participants, predictions from the current models should not be generalized to this group without caution. These representation gaps underscore the importance of collecting more balanced datasets in future research and integrating fairness-aware ML techniques to ensure that predictions are equitable across racial, gender, and other identity-based subgroups. Addressing these issues would strengthen the ability of such models to inform targeted, culturally responsive, and inclusive interventions.

## 5 Limitations and future research

Like other studies, the current study has limitations. First, the findings may not generalize beyond the specific geographic and demographic context of New Mexico. Although our focus on 13 counties with diverse rural and urban populations strengthens the internal validity of findings, the unique sociocultural dynamics of the state—particularly the high proportions of Hispanic and Native American residents—may limit applicability to other regions with similar population structures.

Second, due to the cross-sectional survey design, causal relationships cannot be established. While our models identify strong associations between sociodemographic features and healthcare outcomes, these findings should be interpreted as correlational. Longitudinal or randomized controlled trials are needed to determine whether modifying these predictors would lead to measurable changes in healthcare access or utilization.

Third, although our overall sample size (*N* = 9,099) is adequate, it is relatively modest for machine learning applications. Nonetheless, we limited the number of predictors and used interpretable techniques to reduce the risk of overfitting. We also applied robust model evaluation procedures—including stratified train-test splits, hyperparameter tuning, and validation through cross-validation and, where applicable, out-of-bag error estimation. Despite these precautions, future studies should seek to replicate these findings in larger and more diverse samples.

Fourth, as with all self-reported data, there is potential for measurement error or reporting bias. Respondents may underreport need for or barriers to mental health care, in particular due to stigma, social desirability, or fear. In New Mexico, fear of deportation concerns many families, contributing to lack of reporting concerns and seeking care (48). Our study relied on categorical variables and ordinal scales that do not fully capture the nuance of participants’ experiences. Our study was conducted using data from 2019 to 2024, spanning the COVID-19 pandemic. As very few counties were implementing 100% New Mexico prior to March 2020 and the urgency of the pandemic paused survey activities, the large majority of the data were collected in 2021–2024. It is possible that rates of need for and access to the services in our study differed before and after the pandemic; research shows increases in need and access challenges during the pandemic with some improvements in each sector studied over time (49, 50).

Fifth, while we prioritized interpretability using tools like SHAP values and PDP, these methods still reflect model-driven associations rather than direct explanations or causation. SHAP values, for instance, can vary widely based on model architecture and input distributions, and should not be interpreted as definitive causal effects. Future research could complement model interpretation with qualitative or mixed-methods approaches to contextualize how individuals understand and navigate healthcare access in their daily lives.

Sixth, although our analyses examined each primary outcome domain—mental, medical, and dental care needs and barriers—separately, we did not further stratify results by additional sub-groups (e.g., specific demographic or contextual categories). Future research could expand upon the present findings by conducting such sub-group analyses to explore whether the observed patterns vary across different populations. This work would help tailor interventions to the needs of more narrowly defined groups while building upon the foundation established here.

Seventh, although our models performed well across several metrics, public health utility depends not only on technical performance but also on stakeholder engagement, trust, and integration with existing systems. Future work should explore how interpretable ML models can be implemented in collaboration with community partners, local health departments, and healthcare systems to guide equitable policy development and resource allocation.

Finally, while our study focuses on developing and interpreting predictive models, the translation of such models into routine public health workflows will require careful implementation planning, ideally in collaboration with implementation scientists. Future work could apply established frameworks such as CFIR or RE-AIM (51) to guide integration, adoption, and sustainability within diverse local contexts.

## Data Availability

The data analyzed in this study is subject to the following licenses/restrictions: the data used in this study are not publicly available because individuals who completed the survey did not provide consent for their data to be shared through public access. Access to the dataset may be granted upon reasonable request and with appropriate approvals from the data custodians. Requests to access these datasets should be directed to JM, jmccrae@chapinhall.org.
