# Supplementary material for: A machine learning approach to healthcare needs and barriers using the 100% Community Survey of access to SDOH services
Source: Front Public Health. 2025 Sep 10;13:1659322. doi: 10.3389/fpubh.2025.1659322 (PMC12457340; doi:10.3389/fpubh.2025.1659322)
Supplement: Supplementary file 1 [file Table_1.docx]

Supplementary Material

# Supplementary Tables

Table 1. Feature Importance for Predicting Mental Health Care Needs Using Random Forest.

| **Rank** | **Feature** | **Importance** |
| --- | --- | --- |
| 1 | Caregiver Age | 0.1535 |
| 2 | Family support | 0.1371 |
| 3 | Household income | 0.0991 |
| 4 | Household size | 0.0963 |
| 5 | Gender | 0.0676 |
| 6 | County: Bernalillo | 0.0647 |
| 7 | County: Doña Ana | 0.0557 |
| 8 | Number of children underage 5 | 0.0543 |
| 9 | Race: White | 0.0507 |
| 10 | Number of children aged 5–18 | 0.0499 |
| 11 | Parent of child aged 18 or younger | 0.0427 |
| 12 | Ethnicity: Hispanic | 0.0235 |
| 13 | County: Rio Arriba | 0.0205 |
| 14 | County: Taos | 0.0150 |
| 15 | County: Santa Fe | 0.0135 |
| 16 | Race: Native American | 0.0131 |
| 17 | County: Otero | 0.0087 |
| 18 | County: Socorro | 0.0056 |
| 19 | County: Curry-Roosevelt | 0.0052 |
| 20 | Race: Asian | 0.0045 |
| 21 | County: Valencia | 0.0041 |
| 22 | County: Catron | 0.0040 |
| 23 | County: San Juan | 0.0040 |
| 24 | County: Guadalupe | 0.0033 |
| 25 | Race: Black | 0.0025 |
| 26 | County: San Miguel | 0.0009 |

Table 2. Global Feature Importance for Predicting Medical Care Needs Based on Mean Absolute SHAP Values from the Feedforward Neural Network.

| **Rank** | **Feature** | **Mean Absolute SHAP Value** |
| --- | --- | --- |
| 1 | Household income | 0.0112 |
| 2 | Race: White | 0.0108 |
| 3 | Number of children underage 5 | 0.0100 |
| 4 | Household size | 0.0088 |
| 5 | Parent of child under 18 | 0.0086 |
| 6 | Gender | 0.0071 |
| 7 | Number of children age 5–18 | 0.0064 |
| 8 | Family support | 0.0063 |
| 9 | Caregiver Age | 0.0045 |
| 10 | County: Valencia | 0.0037 |
| 11 | County: San Miguel | 0.0035 |
| 12 | County: Otero | 0.0033 |
| 13 | Ethnicity: Hispanic | 0.0026 |
| 14 | County: San Juan | 0.0017 |
| 15 | County: Guadalupe | 0.0016 |
| 16 | County: Rio Arriba | 0.0016 |
| 17 | County: Curry-Roosevelt | 0.0012 |
| 18 | County: Doña Ana | 0.0012 |
| 19 | County: Catron | 0.0011 |
| 20 | Race: Native American | 0.0010 |
| 21 | County: Taos | 0.0009 |
| 22 | County: Bernalillo | 0.0007 |
| 23 | Race: Black | 0.0005 |
| 24 | County: Socorro | 0.0004 |
| 25 | County: Santa Fe | 0.0004 |
| 26 | Race: Asian | 0.0003 |

Table 3. Global Feature Importance for Predicting Barriers to Dental Care Based on Mean Absolute SHAP Values from the Feedforward Neural Network

| **Rank** | **Feature** | **Mean Absolute SHAP Value** |
| --- | --- | --- |
| 1 | Parent of child under 18 | 0.0198 |
| 2 | Household income | 0.0193 |
| 3 | Gender | 0.0190 |
| 4 | Household size | 0.0134 |
| 5 | Caregiver Age | 0.0096 |
| 6 | Race: White | 0.0085 |
| 7 | County: San Juan | 0.0085 |
| 8 | Number of children underage 5 | 0.0078 |
| 9 | County: Curry-Roosevelt | 0.0066 |
| 10 | County: Santa Fe | 0.0058 |
| 11 | County: Otero | 0.0057 |
| 12 | Family Support | 0.0044 |
| 13 | County: Doña Ana | 0.0040 |
| 14 | Number of children aged 5–18 | 0.0038 |
| 15 | County: Bernalillo | 0.0038 |
| 16 | Ethnicity: Hispanic | 0.0029 |
| 17 | County: Taos | 0.0024 |
| 18 | County: Rio Arriba | 0.0023 |
| 19 | County: Valencia | 0.0023 |
| 20 | County: Socorro | 0.0021 |
| 21 | County: Guadalupe | 0.0018 |
| 22 | County: San Miguel | 0.0015 |
| 23 | Race: Native American | 0.0010 |
| 24 | County: Catron | 0.0009 |
| 25 | Race: Black | 0.0007 |
| 26 | Race: Asian | 0.0005 |

Table 4. Global Feature Importance for Predicting Barriers to Mental Care Based on Mean Absolute SHAP Values from the Feedforward Neural Network.

| **Rank** | **Feature** | **Mean Absolute SHAP Value** |
| --- | --- | --- |
| 1 | Family support | 0.0314 |
| 2 | Gender | 0.0169 |
| 3 | Ethnicity: Hispanic | 0.0161 |
| 4 | County: Bernalillo | 0.0112 |
| 5 | County: Socorro | 0.0105 |
| 6 | Race: White | 0.0101 |
| 7 | Household size | 0.0064 |
| 8 | Household income | 0.0064 |
| 9 | Number of children underage 5 | 0.0056 |
| 10 | Caregiver Age | 0.0055 |
| 11 | County: Taos | 0.0054 |
| 12 | Race: Native American | 0.0051 |
| 13 | County: San Miguel | 0.0040 |
| 14 | Parent of child under 18 | 0.0040 |
| 15 | Number of children aged 5–18 | 0.0039 |
| 16 | County: Curry-Roosevelt | 0.0038 |
| 17 | County: Doña Ana | 0.0033 |
| 18 | County: Valencia | 0.0032 |
| 19 | County: San Juan | 0.0019 |
| 20 | County: Santa Fe | 0.0019 |
| 21 | County: Otero | 0.0009 |
| 22 | County: Rio Arriba | 0.0009 |
| 23 | Race: Asian | 0.0009 |
| 24 | County: Catron | 0.0007 |
| 25 | County: Guadalupe | 0.0005 |
| 26 | Race: Black | 0.0004 |

Table 5. Feature Importance for Predicting Barriers to Medical Care Using the Adaptive Boosting Model

| **Rank** | **Feature** | **Importance** |
| --- | --- | --- |
| 1 | Family support | 0.1385 |
| 2 | Caregiver Age | 0.0949 |
| 3 | Household size | 0.0767 |
| 4 | Number of children aged 5–18 | 0.0735 |
| 5 | Number of children underage 5 | 0.0689 |
| 6 | Household Income | 0.0665 |
| 7 | Ethnicity: Hispanic | 0.0513 |
| 8 | County: Guadalupe | 0.0496 |
| 9 | Gender | 0.0435 |
| 10 | Race: Native American | 0.0429 |
| 11 | County: Taos | 0.0408 |
| 12 | County: Rio Arriba | 0.0369 |
| 13 | County: Bernalillo | 0.0333 |
| 14 | County: Otero | 0.0290 |
| 15 | Race: Black | 0.0266 |
| 16 | County: Curry-Roosevelt | 0.0260 |
| 17 | County: Santa Fe | 0.0241 |
| 18 | County: Catron | 0.0223 |
| 19 | Race: White | 0.0218 |
| 20 | County: San Miguel | 0.0139 |
| 21 | County: San Juan | 0.0116 |
| 22 | Parent of Child Under 18 | 0.0044 |
| 23 | Race: Asian | 0.0030 |
| 24 | County: Socorro | 0.0000 |
| 25 | County: Valencia | 0.0000 |
| 26 | County: Doña Ana | 0.0000 |

Table 6. Feature Importance for Predicting Barriers to Dental Care Using Random Forest

| **Rank** | **Feature** | **Importance** |
| --- | --- | --- |
| 1 | Household income | 0.1469 |
| 2 | Family support | 0.1458 |
| 3 | Household size | 0.0958 |
| 4 | Caregiver Age | 0.0860 |
| 5 | Number of children aged 5–18 | 0.0830 |
| 6 | Number of children underage 5 | 0.0683 |
| 7 | Race: White | 0.0381 |
| 8 | Ethnicity: Hispanic | 0.0377 |
| 9 | County: Taos | 0.0375 |
| 10 | Gender | 0.0330 |
| 11 | Parent of child under 18 | 0.0234 |
| 12 | County: Bernalillo | 0.0210 |
| 13 | County: Doña Ana | 0.0195 |
| 14 | County: Curry-Roosevelt | 0.0194 |
| 15 | County: Santa Fe | 0.0192 |
| 16 | County: Socorro | 0.0186 |
| 17 | County: Otero | 0.0172 |
| 18 | County: San Juan | 0.0169 |
| 19 | County: Valencia | 0.0147 |
| 20 | Race: Native American | 0.0141 |
| 21 | County: San Miguel | 0.0128 |
| 22 | County: Guadalupe | 0.0087 |
| 23 | County: Rio Arriba | 0.0085 |
| 24 | County: Catron | 0.0052 |
| 25 | Race: Asian | 0.0049 |
| 26 | Race: Black | 0.0039 |

Table 7. Performance of All Tested Models Across Healthcare Needs and Barriers

| **Outcome** | **Model** | **Precision** | **Recall** | **Accuracy** | **F1** |
| --- | --- | --- | --- | --- | --- |
| **Mental Health Needs** | Logistic Regression | 0.47 | 0.58 | 0.59 | 0.52 |
|  | Lasso | 0.48 | 0.57 | 0.59 | 0.52 |
|  | **Random Forest** | **0.40** | **0.97** | **0.42** | **0.57** |
|  | XGBoost | 0.48 | 0.53 | 0.60 | 0.51 |
|  | Adaptive Boosting | 0.48 | 0.59 | 0.60 | 0.53 |
|  | K-Nearest Neighbors | 0.48 | 0.50 | 0.60 | 0.49 |
|  | Feedforward Neural Network | 0.64 | 0.13 | 0.64 | 0.22 |
| **Medical Needs** | Logistic Regression | 0.93 | 0.62 | 0.62 | 0.75 |
|  | Lasso | 0.92 | 0.65 | 0.64 | 0.76 |
|  | Random Forest | 0.90 | 0.90 | 0.82 | 0.90 |
|  | XGBoost | 0.91 | 0.80 | 0.75 | 0.85 |
|  | Adaptive Boosting | 0.91 | 0.77 | 0.72 | 0.83 |
|  | K-Nearest Neighbors | 0.90 | 0.85 | 0.78 | 0.88 |
|  | **Feedforward Neural Network** | **0.90** | **0.99** | **0.90** | **0.94** |
| **Dental Needs** | Logistic Regression | 0.92 | 0.60 | 0.60 | 0.72 |
|  | Lasso | 0.89 | 0.71 | 0.67 | 0.79 |
|  | Random Forest | 0.89 | 0.87 | 0.79 | 0.88 |
|  | XGBoost | 0.90 | 0.77 | 0.72 | 0.83 |
|  | Adaptive Boosting | 0.90 | 0.73 | 0.69 | 0.80 |
|  | K-Nearest Neighbors | 0.89 | 0.83 | 0.77 | 0.89 |
|  | **Feedforward Neural Network** | **0.87** | **0.99** | **0.87** | **0.93** |
| **Mental Health Barriers** | Logistic | 0.56 | 0.56 | 0.56 | 0.56 |
|  | Lasso | 0.55 | 0.56 | 0.55 | 0.56 |
|  | Random Forest | 0.55 | 0.59 | 0.55 | 0.56 |
|  | XGBoost | 0.55 | 0.59 | 0.55 | 0.57 |
|  | Adaptive Boosting | 0.56 | 0.58 | 0.56 | 0.57 |
|  | K-Nearest Neighbors | 0.54 | 0.58 | 0.54 | 0.56 |
|  | **Feedforward Neural Network** | 0.52 | 0.94 | 0.53 | 0.67 |
| **Medical Barriers** | Logistic | 0.57 | 0.33 | 0.63 | 0.42 |
|  | Lasso | 0.47 | 0.55 | 0.57 | 0.51 |
|  | Random Forest | 0.49 | 0.60 | 0.58 | 0.54 |
|  | XGBoost | 0.48 | 0.57 | 0.57 | 0.52 |
|  | **Adaptive Boosting** | 0.41 | 0.99 | 0.94 | 0.58 |
|  | K-Nearest Neighbors | 0.47 | 0.59 | 0.56 | 0.52 |
|  | Feedforward Neural Network | 0.54 | 0.37 | 0.61 | 0.44 |
| **Dental Barriers** | Logistic Regression | 0.36 | 0.59 | 0.60 | 0.45 |
|  | Lasso | 0.36 | 0.49 | 0.62 | 0.42 |
|  | **Random Forest** | **0.30** | **0.98** | **0.34** | **0.45** |
|  | XGBoost | 0.38 | 0.42 | 0.65 | 0.40 |
|  | Adaptive Boosting | 0.39 | 0.46 | 0.65 | 0.42 |
|  | K-Nearest Neighbors | 0.33 | 0.52 | 0.57 | 0.40 |
|  | Feedforward Neural Network | 0.78 | 0.01 | 0.72 | 0.02 |
